# Alterations in Autophagic Function and Endoplasmic Reticulum Stress Markers in the Peripheral Blood Mononuclear Cells of Patients on Hemodialysis

**DOI:** 10.3390/ijms26020447

**Published:** 2025-01-07

**Authors:** Wen-Chih Liu, Ming-Yin Wu, Paik Seong Lim

**Affiliations:** 1 Section of Nephrology, Department of Medicine, Antai Medical Care Corporation Antai Tian-Sheng Memorial Hospital, Pingtung 928, Taiwan; wayneliu55@gmail.com; 2 Department of Nursing, Meiho University, Pingtung 912, Taiwan; 3 Department of Biology and Anatomy, National Defense Medical Center, Taipei 114, Taiwan; 4 Division of Renal Medicine, Tungs’ Taichung MetroHarbor Hospital, Taichung 433, Taiwan; ying5430@yahoo.com.tw; 5 Institute of Biomedical Science, College of Life Science, National Chung Hsing University, Taiwan Hospital, Taichung 402, Taiwan

**Keywords:** autophagy, hemodialysis, oxidative stress, endoplasmic reticulum stress

## Abstract

Oxidative stress, endoplasmic reticulum (ER) stress, and alterations in autophagy activity have been described as prominent factors mediating many pathological processes in chronic kidney disease (CKD). The accumulation of misfolded proteins in the ER may stimulate the unfolded protein response (UPR). The interplay between autophagy and UPR in hemodialysis (HD) patients remains unclear. The aim of the present study was to explore the associations between serum oxidative stress markers, autophagy activity, and ER stress markers in the peripheral blood mononuclear cells (PBMCs) of patients on HD. Autophagy and ER stress-related protein expression levels in PBMCs were measured using western blotting. The redox state of human serum albumin was measured via high-performance liquid chromatography. Levels of the microtubule associated protein light chain 3 (LC3)-II, BECLIN1, and p62/SQSTM1 proteins were significantly increased in PBMCs of HD patients compared to healthy subjects. The PBMCs in HD patients also displayed augmented glucose-regulated protein 78 kDa (GRP78), phosphorylated eukaryotic translation initiation factor 2, subunit 1 alpha (*p*-eIF2α), and activating transcription factor 6 (ATF6) levels (*p* < 0.05). Additionally, nuclear factor erythroid 2 (NF-E2)-related factor 2 (NRF2) levels were elevated in the PBMCs of HD patients, compared to those of healthy subjects. Correlation analysis showed that the redox status of albumin was significantly correlated with the p62 protein level in PBMCs. Compared to healthy controls, we found elevated autophagosome formation in HD patients. Increased expression of ER stress markers was also observed in HD patients. Furthermore, increased p62 expression was positively correlated with the protein expression of NRF2, as well as a reduced form of serum albumin (human mercaptalbumin; HMA), in HD patients.

## 1. Introduction

Chronic kidney disease (CKD) has reached epidemic levels worldwide, becoming a major public health challenge over the past decade [1,2]. This condition is associated with increased risks of cardiovascular and infectious mortality, along with structural and functional impairments across various organ systems, particularly the cardiovascular and immune systems. One of the hallmark features of CKD is immune dysfunction, characterized by abnormal activation and impaired function of both the innate and adaptive immune systems. This immune dysfunction contributes to systemic inflammation and oxidative stress, increasing susceptibility to infections. The interplay between these factors may drive the development of autophagy and, in severe cases, apoptosis. While normal autophagy can mitigate oxidative stress and reduce inflammation, dysfunctional autophagy exacerbates these processes.

Autophagy is now known to involve more than 40 Atg proteins that regulate autophagy or autophagy-related processes [3]. Among these, p62 is a key cargo receptor and essential for selective autophagy. These receptors play a pivotal role in forming ubiquitinated protein aggregates for degradation [4,5]. Autophagy is a dynamic and tightly regulated cellular maintenance system that identifies, degrades, and recycles damaged proteins, organelles, and intracellular pathogens [6]. This cytoprotective mechanism, which involves multiple lysosomal enzymes, is essential for cell survival during stress. At baseline, autophagy plays a crucial role in inhibiting oxidative stress and inflammatory responses. However, when autophagy is impaired, toxic metabolites accumulate, potentially contributing to various pathological conditions. Emerging evidence suggests that autophagy is integral to immune function, playing a vital role in both innate and adaptive immunity, and aiding in the direct elimination of pathogens, including bacteria, viruses, fungi, and parasites [7,8,9]. p62, in particular, is a multifunctional protein composed of several functional domains: the N-terminal Phox-BEM1 (PB1) domain, a ZZ-type zinc finger domain, a nuclear localization signal (NLS), a nuclear export signal (NES), an LC3-interacting region (LIR), a KEAP1-interacting region (KIR), and a C-terminal ubiquitin-associated (UBA) domain [10,11].

In parallel, the accumulation of misfolded proteins within the endoplasmic reticulum (ER) triggers the unfolded protein response (UPR) and ER-associated degradation (ERAD), processes collectively known as ER-phagy, which serve to maintain protein homeostasis. ER-phagy eliminates misfolded or unassembled proteins through quality control mechanisms and ensures the fidelity of protein folding [12]. Recent studies have shown that CKD patients exhibit altered autophagy, which may have a protective role against the progression of CKD [13,14,15]. Furthermore, impaired autophagy activation has been linked to cardiac abnormalities in CKD patients [16].

Oxidative stress (OS) occurs when pro-oxidant molecules overwhelm the body’s antioxidant defenses, a state commonly observed in patients undergoing hemodialysis (HD) [17]. Albumin, particularly its reduced form (human mercaptalbumin, HMA), is considered the primary circulating plasma antioxidant [18], while its oxidized form (non-mercaptalbumin, HNA) serves as a reliable indicator of oxidative stress in dialysis patients [19,20,21]. Despite growing awareness of the roles of oxidative stress, ER stress, and autophagy in CKD, the interplay among these processes in HD patients remains poorly understood.

Therefore, the primary objectives of this study were to investigate autophagic activity and ER stress in peripheral blood mononuclear cells (PBMCs) of HD patients and to assess the relationships between albumin redox status, inflammatory markers, and uremic toxins with autophagy and ER stress in this patient population.

## 2. Results

### 2.1. Clinical and Laboratory Findings

The number of normal control (NC) subjects was 10, HD patients without diabetes (non-DM HD) was 17, and HD patients with diabetes (DM HD) was 23. The characteristics of NC, non-DM HD, and DM HD are shown in Table 1. No significant difference was present between the non-DM HD and DM HD groups in terms of age, BMI, triglyceride, hemoglobin, indoxyl sulfate, and LBP. Serum levels of fasting blood sugar (*p* = 0.001), total cholesterol (*p* = 0.009), *p*-cresol (*p* = 0.010), and creatinine (*p* = 0.003) were significantly greater in DM HD compared to non-DM HD. We also did observe a significant difference in HD vintage, with non-DM patients having a longer HD duration compared to DM patients. Neither group of HD and NC had clinical or laboratory evidence of acute or chronic inflammatory diseases based on the serum levels of CRP and IL-6.

### 2.2. Correlation Analyses Between the Clinical and Biochemical Parameters

#### 2.2.1. Autophagy Markers in PBMCs from HD Patients

**Figure 1:**
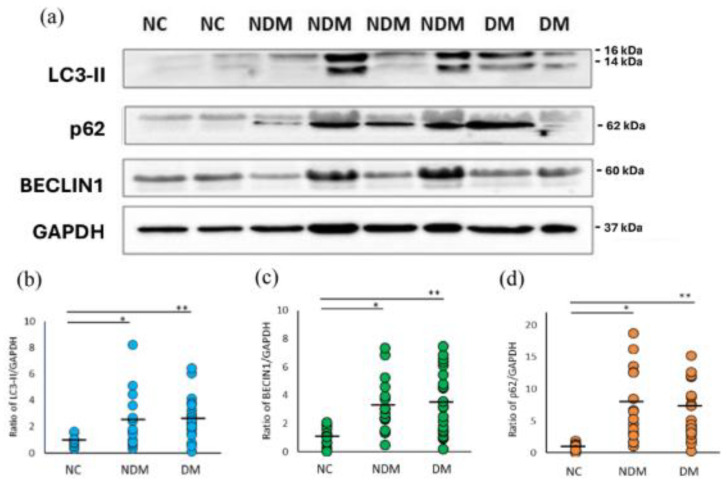
PBMC autophagy levels in NC and HD patients with/without DM. (**a**) Representative Western blot analysis of protein expression. Levels of the autophagy-associated proteins (**b**) LC3-II, (**c**) BECLIN1, and (**d**) p62 were quantified. GAPDH was used as a loading control. The number of NC subjects was 10, non-DM HD was 17, and DM HD was 23. * *p* < 0.05 is NC vs. non-DM HD. ** *p* < 0.05 is NC vs. DM HD. LC3-II, microtubule associated proteins 1A/1B light chain 3A; p62, ubiquitin binding protein p62; NC, normal control; HD, hemodialysis; DM, diabetes; NDM, non-DM.

Autophagosomes can be monitored by the biochemical detection of LC3-II via immunoblotting. Western blot analysis of the LC3-II (Figure 1a,b) indicated that the protein level of LC3-II was significantly increased in PBMCs of HD patients (non-DM and DM), in comparison to NC group (*p* = 0.03 and *p* = 0.003, respectively); however, there were no significant differences between HD patients with and without DM (*p* = 0.76). We noticed the strong band density difference of Berlin-1 (non-DM HD, *p* = 0.002; DM HD, *p* = 0.004) and p62 (non-DM HD, *p* = 0.002; DM HD, *p* = 0.000) between HD patients with NC group (Figure 1a,c,d). In addition, there were no significant differences between HD patients with or without DM in the expression of p62 and Berlin-1 (*p* = 0.82 and *p* = 0.52).

#### 2.2.2. ER Stress and UPR Markers in PBMCs from HD Patients

**Figure 2:**
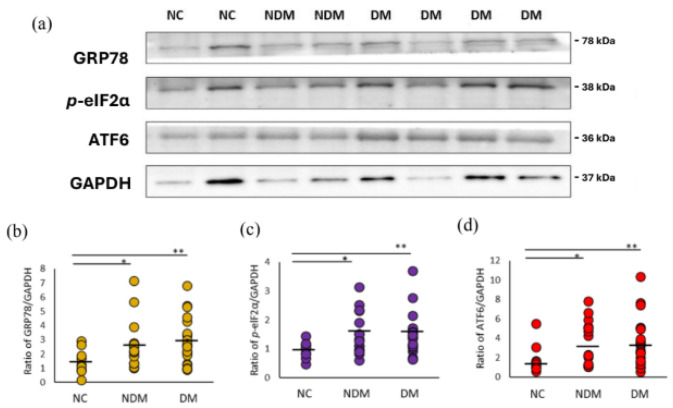
ER Stress and UPR marker levels of PBMCs in NC and HD patients with/without DM. (**a**) Representative western blot images of protein expression. Levels of the autophagy-associated proteins (**b**) GRP 78, (**c**) *p*-eIF2α, and (**d**) ATF6 were quantified. GAPDH was used as a loading control. The number of NC subjects was 10, non-DM HD was 17, and DM HD was 23. * *p* < 0.05 is NC vs. non-DM HD. ** *p* < 0.05 is NC vs. DM HD. GRP78, glucose-regulated protein 78/72 kDa (BiP); *p*-eIF2α, phosphorylated eukaryotic translation initiation factor 2, subunit 1 alpha; ATF6, activating transcription factor 6; NC, normal control; HD, hemodialysis; DM, diabetes; NDM, non-DM.

Furthermore, we checked the following UPR markers: glucose-regulated protein 78 kDa (GRP78), phosphorylated eukaryotic translation initiation factor 2, subunit 1 alpha (*p*-eIF2α), and activating transcription factor 6 (ATF6). We found that the protein expression of GRP78 was significantly higher in PBMCs from the HD patients (about 40% increase), in comparison to the NC group (non-DM HD and DM HD, *p* = 0.049 and *p* = 0.027, respectively) (Figure 2a,b). We observed significant positive correlations with *p*-eIF2α (non-DM HD and DM HD, *p* = 0.033 and *p* = 0.049, respectively) and ATF6 (non-DM HD and DM HD, *p* = 0.036 and *p* = 0.045, respectively) in the HD patients compared to NC subjects (Figure 2a,c,d). In addition, there were no significant differences between HD patients with or without DM in the expression of GRP78, *p*-eIF2α, ATF6 (*p*-values: 0.81, 0.79, 0.96, respectively).

#### 2.2.3. ER Stress Response Transcription Factor (NRF2)

**Figure 3:**
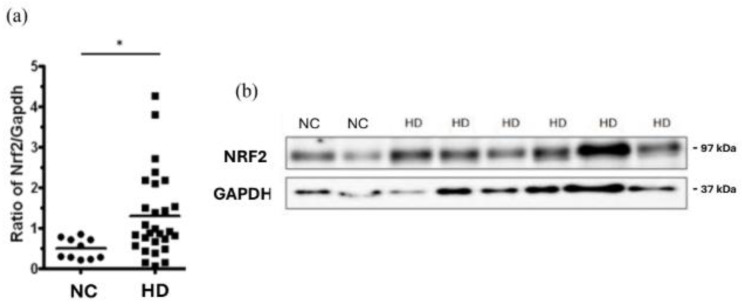
Increased Nrf2 expression in HD patients compared to normal control population. (**a**) NRF2 levels of PBMCs in HD patients and NC subjects. (**b**) Representative western blot analysis of NRF2 protein expression. * *p* < 0.05 is NC vs. HD. NRF2, nuclear factor erythroid 2 (NF-E2)-related factor 2; NC, normal control; HD, hemodialysis.

We also found that the nuclear factor erythroid 2 (NF-E2)-related factor 2 (NRF2) was significantly higher in PBMCs from the HD patients, in comparison with the control group (Figure 3a,b).

### 2.3. Correlation Analyses Between the Clinical and Biochemical Characteristics and Markers Involved in Autophagy in PBMCs

**Figure 4:**
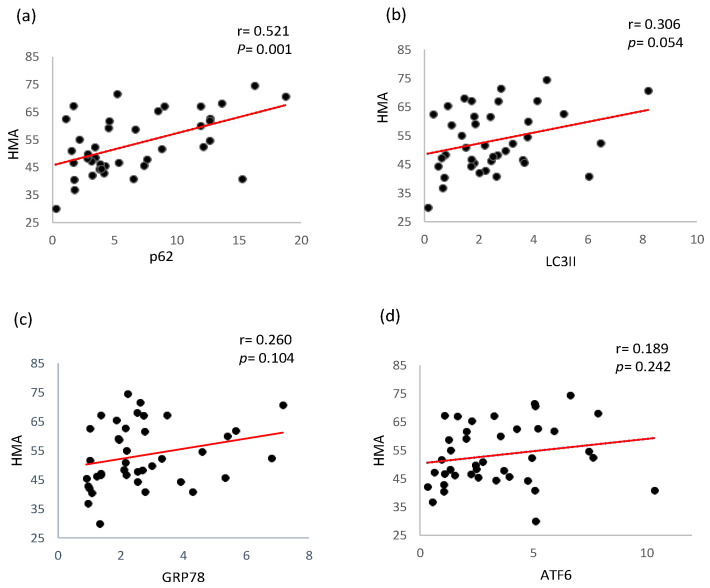
Correlation analysis between HMA and p62, LC3-II, GRP78, and ATF6. The expression of PBMCs in HD patients: (**a**) correlation analysis between HMA and p62 (**b**) correlation analysis between HMA and LC3-II, (**c**) correlation analysis between HMA and GRP78, (**d**) correlation analysis between HMA and ATF6. LC3-II, microtubule associated proteins 1A/1B light chain 3A; GRP78, glucose-regulated protein 78 kDa (BiP); ATF6, activating transcription factor 6. Black dots represent values of the various variables (X-axis). The red line represents the line of best fit.

As depicted in Figure 4, the HMA concentration was significantly correlated with the p62 protein level (Figure 4a, r = 0.471, *p* < 0.002). Similarly, HMA was also weakly positively correlated with the protein expression of LC3-II (Figure 4b, r = 0.306, *p* = 0.054) but not significantly correlated with GRP78 (Figure 4c, r = 0.260, *p* = 0.104) or ATF6 (Figure 4d, r = 0.189, *p* = 0.242).

### 2.4. The Inflammation Maker and Autophagy and ER Stress

**Figure 5:**
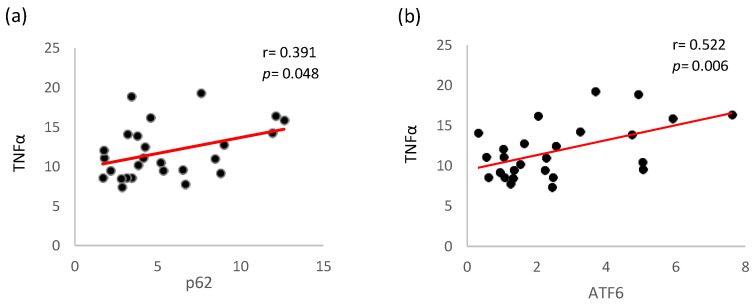
The expression of PBMCs in HD patients: (**a**) correlation analysis between TNFα and p62, (**b**) correlation analysis be-tween TNFα and ATF6. TNFα, tumor necrosis factor alpha; ATF6, activating transcription factor 6.

Moreover, TNFα levels were significantly positively correlated with p62 protein levels (Figure 5a, r = 0.410, *p* = 0.038) and ATF6 expression (Figure 5b, r = 0.391, *p* = 0.048) in PBMCs of the total study population.

## 3. Discussion

The results of our study highlight a strong link between the uremic environment and the upregulation of autophagy and ER stress in dialysis patients. Autophagy is a highly conserved degradation and recycling system in eukaryotes, which is crucial for maintaining cellular homeostasis [12]. We observed elevated levels of key autophagy markers, including LC3-II, BECLIN1, and p62/SQSTM1 in peripheral blood mononuclear cells (PBMCs) of HD patients, when compared to healthy controls. The increased LC3-II levels (Figure 1b), which indicate the abundance of mature autophagosomes, suggest enhanced autophagic activity [22], initially recognized for its role in degrading long-lived proteins and damaging organelles to recycle nutrients and produce energy [23]. This finding is consistent with previous studies that also reported elevated LC3-II levels in HD patients relative to those with CKD or healthy individuals [16]. Additionally, BECLIN1 levels were significantly elevated (Figure 1c), further supporting the hypothesis that autophagy is activated as an adaptive response to metabolic stress. The observed increase in autophagy markers may be attributed to chronic oxidative stress, a common feature of renal dysfunction [24].

Notably, we also observed increased levels of p62 (Figure 1d), a classical autophagy receptor that plays a critical role in selective autophagy through binding to ubiquitinated proteins and facilitating their degradation. p62 is a multifunctional protein involved in several signaling pathways. p62 is an autophagy substrate widely used as a marker for monitoring autophagy activity. Beyond its role as an autophagy substrate, recent studies have revealed that p62 also transports ubiquitinated proteins, such as tau, to the proteasome for degradation [25]. Additionally, p62 can shuttle between the nucleus and cytoplasm, binding ubiquitinated cargo in both compartments to maintain protein quality control [26,27]. This dual functionality positions p62 as a critical link between the ubiquitin–proteasome system (UPS) and the autophagy pathway, facilitating the selective degradation of ubiquitinated proteins [25]. A key finding of our study is the elevated expression of p62 (Figure 1d) in dialysis patients, even in the context of increased autophagic activity.

Through its LC3-interacting region (LIR), p62 binds to LC3-II, enabling the selective transport of ubiquitinated proteins into autophagosomes for degradation. While p62 accumulation is often associated with impaired autophagic flux [28], its downregulation can indicate a highly active autophagic process [29]. p62 identifies and delivers damaged proteins to autophagosomes, facilitating their efficient degradation [30]. This mechanism is crucial for dialysis patients, as persistent cellular stress and protein aggregation present substantial risks to cellular function and viability. Interestingly, the elevated levels of p62 observed in this study align with other reports, suggesting that p62 overexpression can promote the formation of protein aggregates and serve a protective role through enhancing cell survival [24,31]. In contrast, the absence of p62 in cardiomyocytes has been shown to impair the formation of LC3-II, aggresomes, and autophagosomes, leading to exacerbated misfolded protein stress, reduced cell viability, and heightened cellular injury [32]. Thus, the upregulation of p62 in the PBMCs of dialysis patients may reflect an adaptive response to oxidative stress and protein aggregation, driven by the toxic uremic milieu. This finding underscores the importance of p62 in cellular survival mechanisms, particularly under pathological conditions characterized by persistent stress and impaired proteostasis, regardless of the overall autophagic activity.

The UPS is also a primary intracellular degradation pathway to maintain cellular homeostasis. The UPS is responsible for approximately 80% of protein degradation in cells [33] and plays a vital role in the breakdown of damaged, short-lived, and misfolded proteins. Our study also highlights the role of the unfolded protein response (UPR) in HD patients. Markers of the UPR or ER stress, GRP78, *p*-eIF2α, and ATF6, were significantly elevated in PBMCs from HD patients compared to healthy controls. These findings suggest an increased reliance on the UPR to manage the accumulation of misfolded proteins and reduce cellular stress. The UPR promotes proteostasis through the endoplasmic reticulum-associated degradation (ERAD) mechanism [34], which recognizes unfolded proteins in the ER and redirects them to the cytosol for degradation via the UPS [35]. The upregulation of GRP78 (Figure 2b)—a chaperone protein central to the UPR—further supports the presence of chronic ER stress in dialysis patients [36]. GRP78 activation reflects the cell’s ongoing effort to restore protein homeostasis by mitigating the burden of misfolded proteins [37]. Similarly, *p*-eIF2α (Figure 2c) and ATF6 (Figure 2d)—markers of translational repression and stress response protein expression—indicate that cells are reducing global protein synthesis to alleviate ER stress while simultaneously enhancing protective mechanisms [38,39].

Rodrigues et al. demonstrated that endothelial cells exposed to uremic toxins exhibit increased protein carbonylation levels and heightened sensitivity to hydrogen peroxide due to impaired autophagic flux at the lysosomal stage [40]. This impairment inhibits the degradation of oxidized proteins and organelles, leading to their accumulation [41] and rendering cells more susceptible to oxidative stress [42]. Additionally, uremic toxins are shown to promote vascular senescence, suggesting that defective autophagy may contribute to the accelerated vascular aging observed in CKD patients [43]. In our study, we observed elevated markers of oxidative stress, such as GRP78, *p*-eIF2α, and ATF6, in HD patients (Figure 2a–d). These findings are consistent with the notion that functional autophagy is crucial for endothelial cells to mitigate damage induced by uremic toxins.

p62 plays a pivotal role not only in autophagy, but also in regulating the KEAP1/NRF2 pathway [44], which is a critical system for cellular oxidative stress responses. In this pathway, KEAP1 acts as an adaptor protein for the Cullin-3 (Cul3)-type ubiquitin ligase complex, which targets NRF2 for proteasomal degradation under basal conditions. NRF2 is a transcription factor that regulates the expression of numerous antioxidants and cytoprotective genes [45,46]. Under stress conditions, such as exposure to reactive oxygen species (ROS) or electrophilic compounds, KEAP1 functions as a sensor and inhibits NRF2 degradation. This allows NRF2 to accumulate, translocate to the nucleus, and activate the transcription of genes encoding antioxidant proteins and anti-inflammatory enzymes, thereby protecting the cell from oxidative damage. Importantly, p62 contains a KEAP1-interacting region (KIR) which enables it to directly bind to KEAP1. Under oxidative stress conditions, p62 expression is upregulated, partly due to NRF2 nuclear translocation and its activation of target genes. This interaction disrupts KEAP1’s ability to target NRF2 for degradation, further stabilizing NRF2 and creating a positive feedback loop that enhances the cellular stress response. Notably, Nrf2 target genes extend beyond antioxidant proteins and anti-inflammatory enzymes, also encompassing Atg proteins and proteasome subunits, thus contributing to proteostasis [47].

The increased p62 can compete with NRF2 for binding to KEAP1, thus creating a positive feedback loop [48,49]. Our findings demonstrated significantly elevated NRF2 levels in PBMCs from HD patients compared to healthy controls (Figure 3a,b). This suggests that the uremic environment induces NRF2 activation as part of an adaptive response to chronic oxidative stress. The accumulation of p62 may reinforce this response, positioning it as a central regulator of cellular defense mechanisms. This network is particularly vital for dialysis patients, where persistent oxidative stress arises from the accumulation of uremic toxins and metabolic imbalances. These observations are consistent with findings in CKD animal models, where Nrf2 activation led to the upregulation of antioxidant and detoxifying gene products, mitigating oxidative stress and inflammation [50,51]. However, our results contrast with those of Pedruzzi et al., who reported significantly lower Nrf2 mRNA expression in PBMCs from HD patients using low-flux dialyzers, as our dialyzer membrane was ultrapure high flux. This discrepancy could be attributed to differences in dialysis modalities, oxidative stress levels, or study populations, warranting further investigation [52]. Together, our data highlight the importance of the p62–KEAP1–NRF2 axis in managing oxidative stress and proteostasis, especially in the context of the chronic stress conditions experienced by HD patients.

Our data also revealed a significant positive correlation between oxidative stress (as reflected by the serum albumin redox state) and p62 expression in PBMCs from dialysis patients (Figure 4a); specifically, the reduced form of albumin, human mercaptalbumin (HMA), which contains a free thiol group with antioxidant properties, was positively associated with p62 levels. Under normal physiological conditions, HMA constitutes the majority of circulating albumin. However, under oxidative stress conditions, such as those experienced by dialysis patients, albumin is oxidized, leading to a reduced proportion of HMA and increased levels of oxidized albumin [53]. This positive correlation suggests that p62 upregulation in dialysis patients is part of the body’s adaptive response to oxidative stress. While HMA neutralizes reactive oxygen species (ROS) extracellularly [18], p62 functions intracellularly to regulate oxidative stress responses and maintain protein homeostasis. The interplay between these two processes likely explains the observed relationship, with elevated oxidative stress triggering broader cellular responses involving both the antioxidant capacity of HMA and the stress response functions of p62. Together, these mechanisms highlight the critical role of p62 in mitigating oxidative damage and maintaining proteostasis in the challenging uremic environment of dialysis patients.

Interestingly, our study revealed no significant differences in autophagy (LC3-II, p62, Beclin-1; *p*-values: 0.76, 0.52, 0.81, respectively) or ER stress markers (GRP78, *p*-eIF2α, ATF6; *p*-values: 0.81, 0.79, 0.96, respectively) between diabetic and non-diabetic HD patients, suggesting that the uremic environment, rather than diabetes itself, is the primary driver of these alterations. Similarly, there were no notable differences in the differentiation or distribution of T cells between diabetic and non-diabetic HD patients. However, significant correlations were observed between master transcription factors of T cells: T-bet and *FOXP3* in diabetic patients and RORγT and FOXP3 in non-diabetic patients [54]. Wei et al. demonstrated that genetically modified mice lacking autophagy-related genes, such as *Atg5*, *Atg7*, *Beclin-1*, and *PIK3C3-Vps34*, have provided valuable insights into the role of autophagy in regulating Treg cell differentiation [55]. These findings suggest a potential link between autophagy and T cell regulation that warrants further exploration. Future studies involving larger cohorts and more specific markers are needed to better understand the influence of diabetes on autophagy, ER stress pathways, and T cell function in HD patients.

There are several limitations to our study. First, the case-control design precludes causal inferences regarding the relationships between autophagy markers and clinical parameters. Second, we measured a limited number of markers, which may not fully capture the complexity of autophagic and ER stress responses. It needs additional experiments to elucidate the underlying mechanisms, autophagy flux, and ER stress response activity in CKD patients. Third, PBMCs may not accurately represent these processes in other tissues. Finally, we did not perform qRT-PCR to validate the protein expression levels. Future studies should address these limitations by incorporating a broader array of markers and tissue-specific analyses.

## 4. Materials and Methods

### 4.1. Study Design

We enrolled 40 HD patients and 10 healthy volunteers as the control group in this study. To be included in the study, patients must have been at least 20 years old and have been on outpatient HD for at least 3 months. All included patients underwent dialysis three times a week using a polysulfone hollow-fiber dialyzer (Fresenius Polysulfone^®^, Fresenius Medical Care; Bad Homburg, Germany) with surface areas of 1.8 m^2^ and 2.0 m^2^. The bicarbonate-based dialysate was delivered at a bicarbonate level of 34 mEq/L. Patients were excluded if they had malignancy, decompensated liver disease, uncontrolled hypertension, severe obesity (BMI > 35), or were currently on carbamazepine, statins, or immunosuppressive agents. A collaborating physician in the study thoroughly reviewed the medical record for each subject. All subjects provided written informed consent to participate, and the protocol was submitted to the institutional review boards of Tungs’ Taichung Metroharbour Hospital (IRB: Approval Number: 107054, Approval Date: 8 November 2018).

### 4.2. Biochemical Determinations

#### 4.2.1. Blood Sample Collection and Processing

Blood samples for laboratory testing were drawn from the venous end of a vascular access at the beginning of the HD session. Within 30 min after sampling, the remaining blood was centrifuged at 3000× *g* for 10 min, the serum was immediately aliquoted, and frozen at −80 °C until further analysis. The high-sensitivity C-reactive protein (hsCRP) levels were determined via a commercial immunoturbidimetric assay using a Hitachi autoanalyzer (model 7170). The CRP detection limit and interval were 0.1 mg/L and 0.1–500.0 mg/L, respectively. The baseline serum albumin was measured using the bromocresol green method on a Hitachi autoanalyzer (model 7170). Serum levels of cholesterol, triglyceride, and low- and high-density lipoprotein cholesterol were determined via standard laboratory methods.

#### 4.2.2. Measurement of Serum Protein Levels

The lipopolysaccharide binding protein (LBP) was determined from serum samples and controls using standardized enzyme-linked immunosorbent assay (ELISA) methods. It is important to note that serum from normal control subjects was used for intra-assay variation, ensuring the reliability of the results. Commercially available ELISA kits were used to determine IL-6 (R&D Systems Inc., Minneapolis, MN, USA, D6050B), tumor necrosis factor alpha (TNF-α) (R&D Systems Inc., DTA00D), and ATF6 (MyBioSource.com, MBS2127246).

#### 4.2.3. Analysis of Albumin Redox State

The albumin redox state was measured using a previously reported high-performance liquid chromatography (HPLC) method, with some modifications. The HPLC-fluorescence detection (HPLC-FD) system consisted of an AS-8010 autosampler (injection volume, 2 mL per specimen; Tosoh, Tokyo, Japan) and a Model FS-8000 fluorescence detector (excitation wavelength, 280 nm; emission wavelength, 340 nm) with a CCPM double-plunger pump (Tosoh) in conjunction with an SC-8020 system controller (Tosoh). A Shodex-Asahipak ES-502N 7C column (Showa Denko, Tokyo, Japan; 10 × 0.76 cm (inner diameter), dimethylaminoethyl-form for ion-exchange HPLC, column temperature, 35 °C ± 0.5 °C) or, in some instances, two Asahipak GS-520H columns (Asahi Chemical Industry; Kawasaki, Japan; 25 × 0.75 cm (inner diameter), maintained at 32 °C) were used. Linear gradient elution was carried out with an ethanol level increasing from 0% to 5% in 0.05 M sodium acetate and 0.40 M sodium sulfate buffer (pH 4.85; acetate–sulfate buffer) at a flow rate of 1.0 mL/min. Deaeration of the buffer solution was achieved through helium bubbling. Based on the HPLC profiles of HSA obtained from these procedures, the values for each fraction were subjected to numerical curve fitting, and the fractions of HMA, HNA-1, and HNA-2 relative to total HSA were calculated.

#### 4.2.4. Isolation of PBMCs

Peripheral blood mononuclear cells (PBMCs) were isolated from whole blood using gradient density centrifugation with Ficoll Paque Plus. Two 5 mL blood samples were carefully layered over 5 mL of Ficoll Paque Plus in sterile 15 mL centrifuge tubes. The samples were centrifuged at 800× *g* for 15 min at 20 °C. The PBMC layer was carefully collected and washed three times with sterile phosphate-buffered saline (PBS), centrifuging each wash at 250× *g* for 10 min at room temperature. The final PBMC pellet from both tubes was combined and stored at −80 °C until further analysis with western blotting.

#### 4.2.5. Western Blot Analysis of PBMC Protein Expression Levels

PBMCs were lysed using RIPA lysis buffer (ab152163, Abcam, Cambridge, UK), and the protein concentrations were determined using the BCA Protein Assay Kit (ab102536, Abcam, Cambridge, UK), following the manufacturer’s instructions. Equal amounts of protein (20 μg per well) were separated using 12% SDS-PAGE. The separated proteins were then transferred onto nitrocellulose membranes at 300 mA for 60 min. To block non-specific binding, the membranes were incubated with Tris-buffered saline containing 0.1% Tween 20 (TBST) and 5% non-fat milk for 2 h at room temperature. After blocking, the membranes were incubated with primary antibodies against microtubule-associated protein 1A/1B light chain 3A (LC3), ubiquitin-binding protein p62, BECLIN1, and GAPDH overnight at 4 °C with gentle shaking. Following primary antibody incubation, the membranes were washed three times with TBST and incubated with appropriate secondary antibodies for 1 h at room temperature. After further washing, the membranes were exposed to ECL substrate, and chemiluminescent signals were detected using the Image Lab™ software (https://www.bio-rad.com/en-ie/product/image-lab-software?ID=KRE6P5E8Z accessed on 1 June 2018). The band densities were quantified using the software’s built-in analysis tools.

The primary antibodies were used at dilutions of 1:1000 for LC3-II (Cell Signaling Technology, Danvers, MA, USA, 12741s), 1:1000 for p62 (Cell Signaling Technology, 5114s), 1:1000 for BECLIN1 (Cell Signaling Technology, D40C5), 1:1000 for GRP78 (Abcam, Cambridge, UK, ab21685), 1:1000 for *p*-eIF2α (Cell Signaling Technology, 9127s), 1:1000 for ATF6 (Invitrogen, Waltham, MA, USA, 70B1413), 1:1000 for nuclear factor-erythroid 2 (NF-E2)-related factor 2 (NRF2) (Cell Signaling Technology, 12721s), and 1:3000 for GAPDH (Cell Signaling Technology, D16H11). After washing with PBST three times, the membranes were treated with HRP-conjugated secondary antibodies for 2 h, and peroxidase-conjugated goat anti-mouse (Cell Signaling Technology, 91196) and goat anti-rabbit (Cell Signaling Technology, 98164) were used.

It is important to recognize that variability in GAPDH levels—a commonly used loading control—may affect the interpretation of protein expression data. To address this, we rigorously verified the normalization process to ensure accurate quantification of target proteins. Minor fluctuations in GAPDH expression were noted, but their impact on data interpretation was minimized through careful normalization. This approach ensures the reliability and consistency of the final analysis of target protein levels. The variability in GAPDH levels observed could arise from both technical and biological factors. Technically, minor inconsistencies in protein extraction efficiency, sample handling, or pipetting during the experimental process could introduce variability. Biologically, it is known that GAPDH expression may fluctuate under specific experimental conditions or disease states, as reported in previous studies [56,57]. Therefore, despite limitations, the normalization strategy implemented here provided a reliable basis for interpreting the data. We recommend incorporating additional controls, such as total protein staining or multiple housekeeping proteins, to enhance the robustness of normalization strategies in western blot analysis.

### 4.3. Statistical Analysis

Data are presented as the mean value ± the standard error of the mean. The chi-square test was used to evaluate the difference in proportions between the two groups. The assumption of normality was tested for each continuous variable. The difference between the two groups was evaluated with a t-test or, in the case of lack of normality, with the Mann–Whitney U-test, and the correlations were tested using Pearson or Spearman correlation coefficients, respectively. The *p*-value < 0.05 was considered statistically significant. All statistical analyses were conducted in R (R version 4.3.2, http://www.r-project.org accessed on 9 September 2024).

## 5. Conclusions

In summary, the elevated levels of p62 and related markers (LC3-II, BECLIN1, GRP78, *p*-eIF2α, and ATF6) in dialysis patients highlight the critical role of p62 as a central regulator of cellular stress responses. p62 coordinates the autophagic and UPS pathways, allowing cells to manage increased protein damage and oxidative stress. Moreover, the positive correlation between p62 and HMA, indicates a link between these two factors, highlighting the important role of p62 in reducing the oxidative burden. However, the persistent elevation of these markers suggests that the degradation systems may be overwhelmed, leading to a potential accumulation of damaged proteins and cellular dysfunction. Further studies with a larger patient cohort and the inclusion of a broader range of autophagic and UPS markers are needed to clarify the mechanisms driving these processes and their impacts on patient health.

## Figures and Tables

**Table 1:** Demographic and baseline characteristics of the study population. Data are expressed as mean ± SD for parametric data or as median (25th and 75th percentiles) for non-parametric data. Means were compared using Student’s *t*-test for normally distributed samples and the Mann–Whitney U-test for non-normally distributed samples. The chi-square test was used to compare proportions among non-DM HD and DM HD groups.

Parameters	Normal Control	Non-DM HD	DM HD	*p* Value
Number (n)	10 (M = 5, F = 5)	17 (M = 10, F = 7)	23 (M = 13, F = 10)	0.884
Age, years	54.9 ± 7.9	58.1 ± 9.0	61.1 ± 9.7	0.268
Vintage, months	-	82.5 ± 51.5	51.5 ± 43.1	0.044 *
BMI ^1^, kg/m^2^	-	24.5 ± 3.5	24.3 ± 3.6	0.788
HTN ^2^ n (%)	0	7 (41.2)	10 (43.5)	0.888
Smoking n (%)	0	6 (35.3)	8 (34.8)	0.974
WBC ^3^, 10^9^/L	5.86 ± 1.04	5.95 ± 1.60	6.30 ± 1.58	0.137
Hb ^4^, g/L	14.5 ± 1.5	11.2 ± 1.7	11.8 ± 1.4	0.290
FBS ^5^, mg/dL	101.3 ± 14.1	91.6 ± 28.1	136.4 ± 43.1	0.001 *
Cr ^6^, mg/dL	0.70 ± 0.27	11.8 ± 1.4	10.0 ± 2.0	0.003 *
T Chol ^7^, mg/dL	205.1 ± 34.6	178.03 ± 2.9	146.9 ± 37.9	0.009 *
TG ^8^, mg/dL	124.9 ± 65.1	155.8 ± 134.9	155.19 ± 5.1	0.985
hsCRP ^9^, mg/dL	-	3.08 ± 3.18	3.153 ± 0.46	0.948
il-6 ^10^, pg/mL	-	4.69 ± 3.65	5.37 ± 3.98	0.582
TNFα ^11^, pg/mL	-	11.06 ± 3.04	12.39 ± 3.61	0.320
LBP ^12^, μg/mL	-	17.46 ± 0.78	16.003 ± 0.93	0.453
HNA ^13^, %	-	43.0 ± 11.1	48.5 ± 11.9	0.145
IS ^14^, mg/dL	-	20.92 ± 11.40	22.51 ± 9.67	0.646
*p*-Cresol, mg/dL	-	10.465 ± 0.45	17.99 ± 11.66	0.010 *

^1^ Body mass index; ^2^ Hypertension; ^3^ White blood cells; ^4^ Hemoglobin; ^5^ Fasting blood sugar; ^6^ Creatinine; ^7^ Total cholesterol; ^8^ Triglyceride; ^9^ High-sensitive C-reactive protein; ^10^ Interleukin 6; ^11^ Tumor necrosis factor alpha; ^12^ Lipopolysaccharide binding protein; ^13^ proportion of serum oxidized albumin; ^14^ Indoxyl sulfate. “*” means *p*-value < 0.05 when comparing non-DM HD group and DM-HD group.

## Data Availability

The data of this study are available by request from the authors.
